# Deep Red Tunability and Output Power Performances of Visible Laser Emission in a Pr:Ba(Y_1−*x*_Lu_*x*_)_2_F_8_ Single Crystal

**DOI:** 10.3390/ma13204655

**Published:** 2020-10-19

**Authors:** Eugenio Damiano, Giovanni Cittadino, Alberto Di Lieto, Mauro Tonelli

**Affiliations:** 1 MEGA Materials s.r.l., Largo Bruno Pontecorvo 3, 56127 Pisa, Italy; g.cittadino@megamaterials.it (G.C.); a.dilieto@megamaterials.it (A.D.L.); m.tonelli@megamaterials.it (M.T.); 2 NEST, Istituto Nanoscienze–CNR, Piazza S. Silvestro 12, 56127 Pisa, Italy; 3 Dipartimento di Fisica, Università di Pisa, Largo Bruno Pontecorvo 3, 56127 Pisa, Italy

**Keywords:** solid-state lasers, visible lasers, single-crystal fibers, spectroscopy, crystal growth, diode-pumped lasers, laser materials, praseodymium-based lasers

## Abstract

The demand for tunable visible laser sources with high power and high beam quality, for application ranging from metrology to remote sensing, is constantly increasing. In this work, we report on the details of crystal growth, via the Czochralski method, and laser characterization of a Pr-doped Ba(Y${}_{1-x}$Lu${}_{x}$)${}_{2}$F${}_{8}$ (BYLF) single crystal, which is a promising candidate for fulfilling these requirements, both in terms of tunability and high-power capabilities. We measured for the first time the laser tunability curve in the deep red region obtaining a continuous range of 17 nm. The laser emission of the three main Pr${}^{3+}$ lines in the visible (orange, red, and deep red) was tested under increased pump power with respect to previous studies on this material, demonstrating output powers of more than 360 mW and no thermal rolloff, up to 1.9 W of absorbed power.

## 1. Introduction

The development of compact, reliable, long-life solid-state visible laser sources with a high output beam quality (nearly TEM${}_{00}$) has become of utmost importance in recent decades due to the countless applications they have in fundamental research, medicine and surgery, entertainment, environmental monitoring, and metrology [1,2]. In particular, in the field of metrology, they have two important features when compared with commercially-available diode lasers. Solid-state lasers have a reduced emission linewidth (at least two orders of magnitude narrower than that emitted by diode lasers). Furthermore, the emission wavelength can be widely tuned. The combination of these two properties make these devices particularly appealing for the excitation of the transitions of ${}^{87}$Sr${}^{0}$ and ${}^{88}$Sr${}^{+}$ for the development of compact and reliable optical atomic clocks [3,4].

The most promising active ion for visible laser emission is certainly praseodymium, given the multitude of transition accessible, and the possibility to pump it with inexpensive blue laser diodes. However, visible laser emission from praseodymium in the most common oxide materials was demonstrated to be inefficient, due to the large phonon energy of this class of compounds. In contrast, in fluorides, visible laser emission was extensively demonstrated in several different crystal hosts [5,6,7,8]. In general, fluorides have worse thermomechanical properties compared to oxides that can limit the output power available under strong optical pumping [9]. Nonetheless, the power scaling in some fluorides has been studied with encouraging results [10].

In this work we further investigate a relatively new monocrystal: Ba(Y${}_{1-x}$Lu${}_{x}$)${}_{2}$F${}_{8}$ (BYLF). This crystal host was introduced as an isomorph of BaY${}_{2}$F${}_{8}$ (BYF). BYF has interesting properties for laser applications, such as narrow phonon spectra with a cut-off energy of 350 cm${}^{-1}$ combined with the fact that it is one of the crystals with the highest Stark splitting [11]. In addition, the symmetry of the crystalline structure and symmetry of the dopant ion site allow us to carry out laser operations not obtained before using BYF, especially for the praseodymium doping case [12]. Moreover, BYF doped with different rare-earth ions has been used in wide laser tunability experiments [13] as well as in laser operation under strong pumping, given its thermal lens compensation properties [14].

All these properties are also present in BYLF [15,16]. However, in comparison to BYF, BYLF shows greater resistance to fractures. In fact, a main issue for BYF is the tendency to be fragile along cleavage planes, making harder the polishing of laser samples and the available volume from the as-grown boule. Enhanced laser performances have also been demonstrated [12]. Although there are no data available on the thermomechanical properties of BYLF, similarities can be spotted with the case of LiYF${}_{4}$ and LiLuF${}_{4}$, in which the substitution of yttrium with lutetium improve thermomechanical properties and high power behavior in high-power laser operation [9,17].

In this paper we describe the growth of single-crystal Pr:BYLF and present interesting results on deep-red wavelength tunability and visible laser output power performances in the orange, red, and deep red lines of Pr${}^{3+}$. In the tunability experiment, a 17 nm uninterrupted tunability in the deep red region was obtained. Under increased optical pumping, with respect to previous studies, the laser emission in the three aforementioned laser lines showed no thermal roll-off or saturation effect, and slope efficiencies greater than 23% and output power greater than 360 mW were obtained for all three lines.

## 2. Materials and Methods

### 2.1. Crystal Growth and Structure

A boule of Pr-doped Ba(Y${}_{1-x}$Lu${}_{x}$)${}_{2}$F${}_{8}$ with x=0.2 was grown via the Czochralski technique, using a custom-made furnace in Pisa University. The 5N-purity raw powders (from AC Materials, Tarpon Springs, FL, USA) of BaLu${}_{2}$F${}_{8}$ and BaY${}_{2}$F${}_{8}$ were mixed in a 20:80 molar proportion in a platinum crucible. We then added praseodymium fluoride (PrF${}_{3}$) to the mixture, to obtain a 1.25% doping level, and barium fluoride (BaF${}_{2}$), to preserve the stoichiometric ratios.

Special care was taken to avoid contamination such as oxygen complexes and, especially, OH${}^{-}$ radicals. For this reason, the whole weighing process was executed in a home-made dry-box with controlled humidity and utilizing a precision scale. In addition to that, an apposite vacuum system dedicated for the furnace was employed to reach a pressure limit below 10${}^{-5}$ Pa. Before starting the growth by dipping a seed of undoped BYLF, an accurate cleaning-procedure of the surface of the melt was performed. The procedure consisted on successive controlled micro-crystallization steps. This important technique allows one to avoid inclusions/defects during the formation of the crystal, minimizing the fluctuation in diameter due to a fast crystal diameter enlargement caused by rapid nucleation from the aforementioned particles.

The growth was performed in a static high-purity (5N) argon atmosphere at a temperature of about 1000 ${}^{\circ }$C. The vertical pulling rate was set at 0.5 mm/h with a rotation rate of 5 rpm.

The furnace is equipped with an optical system to control the diameter during the growth process. A feedback loop allows for the automatic adjustment of the temperature to enlarge or shrink the crystal diameter.

**Figure 1:**
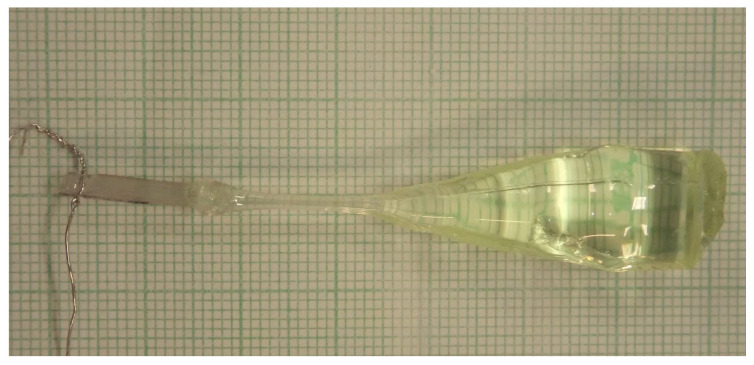
As-grown Pr:BYLF (Ba(Y${}_{1-x}$Lu${}_{x}$)${}_{2}$F${}_{8}$) boule.

As can be seen in Figure 1 the as-grown boule had a transparent and smooth appearance. The thin ‘neck’ is crucial to prevent defects from propagating from the seed into the bulk crystal. After the growth, the sample was visually checked using a microscope and a red laser diode at 680 nm. This procedure allows for the verification of the presence of defects and cracks (dimensions as small as few μm) that, in this case, were absent. This demonstrates the goodness of the growth system and the success of the necking procedure, especially for Pr-doped crystals growths.

BYLF is isomorphic to BYF with unit cell parameters a = 6.94(4)Å, b = 10.57(5)Å, c = 4.27(2)Å, and β = 99.9(5)${}^{\circ }$ (the angle between the a-axis and the c-axis) [18]. As a consequence of being monoclinic, BYLF is a biaxial crystal. The crystallographic axes (a, b, and c) of the boule were identified by means of an X-ray Laue chamber and, subsequently, the optical (indicatrix) axes *x*, *y*, and *z* were identified. Spectroscopy and laser samples were cut from the boule along the *x*-*y*-*z* axes. The specimen faces were optically polished for laser applications. Subsequently, all spectroscopic and laser parameters were measured and here reported along these axes [16].

### 2.2. Spectroscopic Characterization

The polarized ground state absorption relative to the relevant levels for optical pumping, was measured with a Varian Cary 500 integrated spectrophotometer, between 430 and 490 nm, with a resolution of 0.15 nm.

The absorption peak corresponding to the ${}^{3}$H${}_{4}$→${}^{3}$P${}_{2}$ transition was pumped to evaluate the fluorescence spectra in the visible part of the spectrum. The pump was focused on the sample with a focal length f${}_{1}$ = 100 mm lens and the emission was collected by a f${}_{2}$ = 75 mm lens and sent to the entrance of a Jobin–Yvon TRIAX monochromator (Focal length = 33 cm) with a resolution of 0.15 nm. The light was filtered by a polarizer, detected by an Hamamatsu R943-02 phototube, and amplified using the lock-in technique. By changing the orientation of the sample and polarizer, we explored all polarization parallel to the optical axes of the crystal.

### 2.3. Deep-Red Tunability

**Figure 2:**
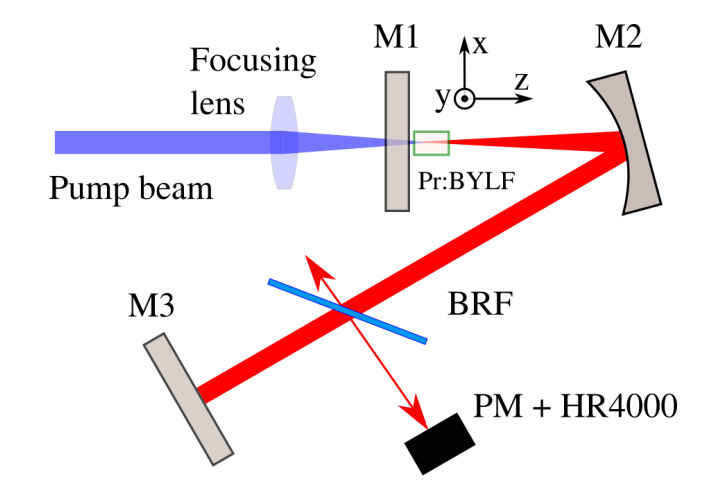
Scheme of laser cavity for tunability measurements. The optical axes system relative to the sample is indicated.

The setup for the tunability in the deep-red region consisted of a 3-mirror V-shaped resonating cavity, shown in Figure 2. The mirror input coupler M1 was a flat reflector, with high reflectivity (>99.5%) between 465–750 nm, and anti-reflective coated at the pump laser wavelength (350–560 nm). The folding mirror M2 was curved, with a radius of curvature of 100 mm, placed at 100 mm from the input coupler, in order to generate a collimated beam in the second arm. Finally, the cavity was closed with a flat mirror M3. The angle between the two arms of the resonator was kept as small as possible, namely 10${}^{\circ }$, to minimize the astigmatism of the laser beam resonating in the cavity.

A sample of Pr:BYLF with a clear aperture of 5.4 × 6.5 mm${}^{2}$ (along the x and y optical axes respectively) and length of 4.3 mm, was employed in the experiments. The sample was carved from the boule and polished on all 6 facets. It was placed near to M1, encased in a custom made copper holder with recirculating water at 16 ${}^{\circ }$C. The z-axis was placed parallel to the resonator axis, in order to excite and observe the x-y plane. The pump source was a single InGaN-based blue laser diode at 445 nm, with maximum power of 1.9 W, emitting light polarized along the y-axis of the sample. In this configuration the absorption efficiency was measured to be η= 70.5%. In order to evaluate the size of the pump beam inside the crystal, a WinCamD-UCD15 CCD beam imaging camera by DataRay (Redding, CA, USA) was placed inside the cavity instead of the sample holder. The pump beam was suitably filtered to avoid damaging the apparatus. The beam radius at the waist position was measured to be $w_{0x}$ = 110 μm and $w_{0y}$ = 45 μm for the Ox and Oy direction, respectively.

An uncoated 2-mm-thick birefringent plate (BRF) was used as a tuning element in the cavity. It was mounted on a precision rotary stage and placed at Brewster’s angle in the collimated arm of the cavity (M2-M3). The relative orientation of the plate and sample allowed us to measure the tunability along the y axis of BYLF. Both M2 and M3 were highly reflective in the laser wavelength range (>99.5%). Due to the lack of suitable output couplers, the reflection from the uncoated birefringent plate was used to evaluate the output wavelength and to get an estimation about the output power. The reflected fraction of the resonating beam was sent alternatively to an Ocean Optics HR4000 portable spectrophotometer (with resolution 1 nm) and to a photodiode powermeter (PM) to perform the two measurements.

### 2.4. Output Power Performances

**Figure 3:**
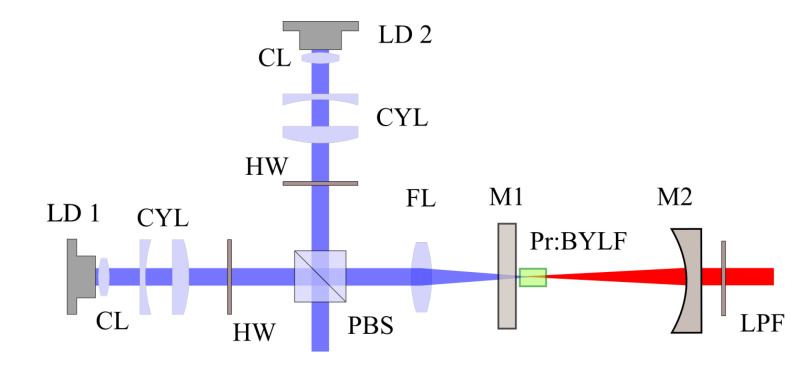
Scheme of laser cavity for laser operation experiment.

We tested the laser efficiency under increasing pump power to identify the effect of possible detrimental thermal effects. To do so we converted the cavity to a simple hemispherical cavity and increased the pump power, by combining two orthogonally polarized laser diodes, as shown in Figure 3.

The two diodes (LD 1 and LD 2), emitting at 445 nm with perpendicular polarization were collimated, conditioned with a pair of cylindrical lenses (CYL), and combined with a polarizing beam splitter (PBS). The combination of the 2 beams was focused on the sample using a f = 40 mm lens. The sample was the same employed in the tunability experiment, with the same orientation. The pump sources had a maximum output power of 1.9 W along *x* and 1.5 W along *y*. The absorption efficiencies for light polarized along the *x* and *y* axes, were $\eta_{x}$ = 60.3% and $\eta_{y}$ = 70.5%. The IC (M1) was the same for the previous experiment, while the output couplers (M2) were changed to achieve several emissions in orange, red, and deep red. The sample was kept in the same copper holder of the tunability test, and placed near to M1. The reflectivity of the mirror used is reported in the result section. This setup reproduces the condition reported in [12], to allow an easier comparison when the input power is increased.

## 3. Results and Discussion

### 3.1. Spectroscopic Characterization

**Figure 4:**
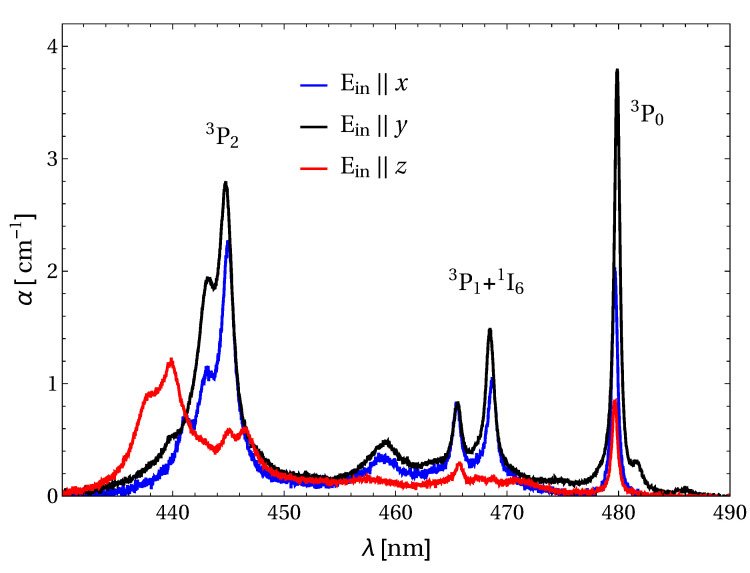
Polarized absorption spectra in Pr:BYLF.

The results of the absorption measurement, with light polarized along the indicatrix axes, are shown in Figure 4.

In the spectra, two almost-equally-intense absorption lines can be observed at 445 nm on the E‖x and E‖y polarizations, corresponding to the ${}^{3}$H${}_{4}\to^{3}$P${}_{2}$ transition. Their peak value between 2 and 3 cm${}^{-1}$ and their relatively large width of several nm (at room temperature), make them suitable for efficient optical pumping with InGaN-based laser diodes. This fact is an advantage since it can be exploited to simultaneously end pump this material on the x-y facet. The spectra also shows the manifolds corresponding to the transition ${}^{3}$H${}_{4}$→${}^{3}$P${}_{1}$ + ${}^{1}$I${}_{6}$ and ${}^{3}$H${}_{4}$→${}^{3}$P${}_{0}$. The latter, located at about 480 nm, could be also exploited for optical pumping for the transitions in the visible, although with reduced efficiency due to the three-level laser scheme obtained.

**Figure 5:**
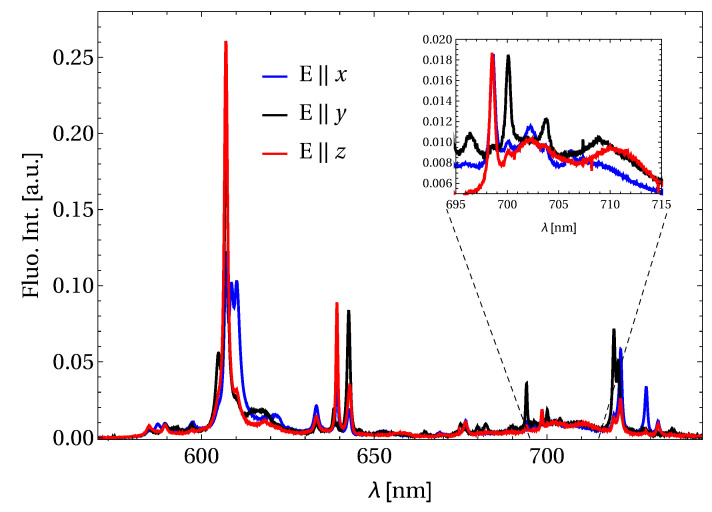
Polarized fluorescence spectra in Pr:BYLF. The insets shows a magnification of the region of interest for deep-red tunability.

Figure 5 reports the fluorescence spectra of Pr:BYLF. In the spectra, several transition lines can be observed in the region ranging from 580 nm to 750 nm related to the ${}^{3}$P${}_{0}$ energy level. A multi-phonon decay allows populating the ${}^{3}$P${}_{0}$ level from the pumped ${}^{3}$P${}_{2}$ one. From this last manifold, three different transitions were detected: ${}^{3}$P${}_{0}\to^{3}$H${}_{6}$ with a strong emission peak at 607 nm, ${}^{3}$P${}_{0}\to^{3}$F${}_{2}$ with tho peaks at 639 nm, and at 643 nm, and ${}^{3}$P${}_{0}\to^{3}$F${}_{4,3}$ with emission peaks around 720 nm.

It worth noticing that a large pedestal (ranging from approximately 690 nm to 720 nm) can be spotted in the deep red region, enabling the possibility of wavelength tuning in this region.

### 3.2. Deep Red Tunability

**Figure 6:**
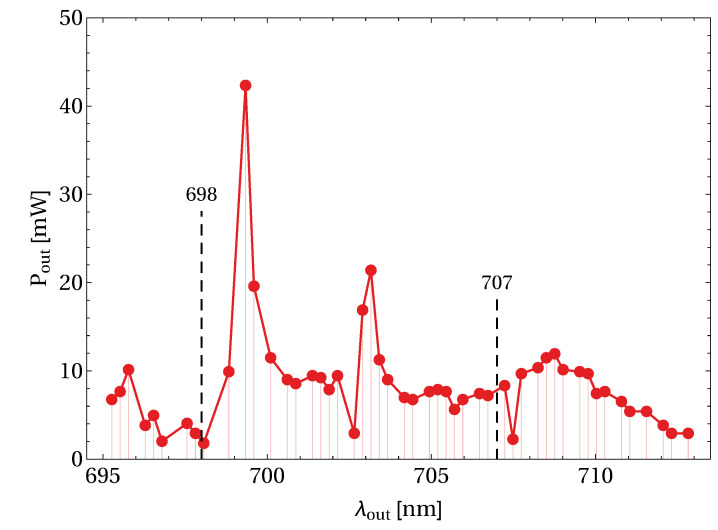
Uninterrupted tunability curve in the deep red in a Pr:BYLF sample, along the E‖y polarization.

The tunability curve we obtained is shown in Figure 6. An uninterrupted tuning range of 17 nm was obtained between 696 and 713 nm in the deep red region. The relatively low power values reported in the graph are due to the very low and parasitic extraction. It is important to notice the fact that the obtained range includes the 698 nm line, corresponding to the clock transition of Sr${}^{0}$, and the 707 nm line, another wavelength required in strontium-based metrology. These results extend the tunability respect to Pr:BYF, which also presented some interruptions around 700 nm [13], not observed in this work in Pr:BYLF.

### 3.3. Output Power Performances

As seen in Figure 5 and as reported in [12], in the 640 nm region there are two equally intense fluorescence lines, located at 639 nm (E‖x,z) and at 643 nm (E‖y) respectively. In the configuration used in this experiment, a competitive behavior was observed between these two lines in the x and y polarizations. Ultimately, the 643 nm line (E‖y) was stabilized and studied.

**Figure 7:**
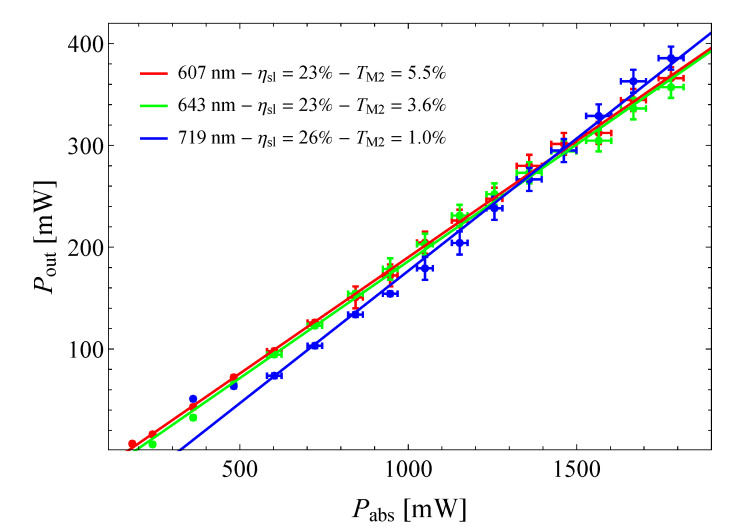
Output powers as a function of absorbed power in laser operation under increased pump power on Pr:BYLF.

The results for laser operation are reported in Figure 7, in which it is shown that the behavior of the output power as a function of absorbed pump power, for three transitions in the orange, red, and deep red. The fitting of the data points indicated a perfectly linear behavior up to 1.9 W of absorbed power, corresponding to 3.4 W of incident power. This is an indication of the good capabilities of Pr:BYLF of sustaining intense optical pumping.

With such pumping power, we obtained maximum output powers of 366 mW, 357 mW, and 386 mW for the transition in the orange at 607 nm, in the red at 643 nm, and in the deep red at 721 nm respectively. These results are remarkable and are on par with others obtained in Pr-doped fluorides in similar conditions [10].

In terms of slope efficiency relative to the absorbed power, we obtained 23% both in the orange and red, and 26% in the deep red. These results match the previously reported ones [12], in terms of slope efficiency and threshold power, extending the explored region in terms of absorbed power from 0.8 W to 1.9 W.

Laser operation results are summarized in Table 1, in which are reported, for the transitions under investigation, the measured laser emission wavelength ($\lambda_{out}$), the transmittance of the output coupler M2 (T${}_{OC}$), the polarization of the output beam with respect to the indicatrix axes (E${}_{out}$), the slope efficiency ($\eta_{sl}$), the threshold power (P${}_{thr}$), and the maximum output power (P${}_{out}$).

## 4. Conclusions

In this paper we reported the first demonstration of wavelength tunability in the deep red region in a Pr:BYLF single crystal, further demonstrating the attractiveness of this material as a robust and reliable active media for visible laser emission. A tunability of 17 nm in the deep red was measured, and this range includes two of the five deep-red wavelengths needed for strontium based metrology. Moreover, the laser emission output in the red, orange and deep-red regions was demonstrated to be linear up to 1.9 W of absorbed power, reaching a maximum output power of about 0.3 W for all three transitions. This extends the previous characterization of the present laser material. These two results combined prove the Pr:BYLF could be used in extremely precise fields like metrology, but at the same time is capable of delivering a fair amount of output power without any thermal rollover.

Both the laser cavities presented could be further optimized to match future requirements in terms of linewidth and output power. This could be done by employing custom-made output couplers and intra-cavity elements such as ethalons. Moreover, future developments in the semiconductor laser technology will also allow us to pump the crystals with better and more brighter beams, improving the overall efficiency of the present class of devices. Finally, the potential of solid-state lasers for miniaturization, will make them competitive with respect to the current laser technologies, enabling the development of long-lasing and reliable laser sources.

## Figures and Tables

**Table 1:** Laser parameters for the three transitions under investigations in the laser experiment on Pr:BYLF.

$\lambda_{\mathrm{out}}$	T${}_{\mathrm{OC}}$	E${}_{\mathrm{out}}$	P${}_{\mathrm{thr}}$	$\eta_{\mathrm{sl}}$	P${}_{\mathrm{out}}$
607 nm	5.5%	‖y	93 mW	23%	366 mW
643 nm	3.6%	‖y	110 mW	23%	357 mW
721 nm	1.0%	‖x	102 mW	26%	386 mW
